# Brivaracetam in Combination with Midazolam and Ketamine Reduces Soman-Induced Seizure and Neurodegeneration in Rats

**DOI:** 10.3390/neurolint18080146

**Published:** 2026-07-30

**Authors:** Lucille A. Lumley, Hailey G. Steier, Sabrina Y. Orta, Donna A. Nguyen, Michael F. Stone, Caroline R. Schultz, Jerome Niquet, Marcio de Araujo Furtado, Claude G. Wasterlain

**Affiliations:** 1 Neuroscience Department, U.S. Army Medical Research Institute of Chemical Defense (USAMRICD), Aberdeen Proving Ground, Aberdeen, MD 21010, USA; 2 Department of Neurology, David Geffen School of Medicine at UCLA, Los Angeles, CA 90095, USA; 3 Epilepsy Research Laboratory (151), Veterans Affairs Greater Los Angeles Healthcare System, Los Angeles, CA 90073, USA; 4 BioSEaD, LLC, Rockville, MD 20850, USA

**Keywords:** antiseizure medication, organophosphorus nerve agent, cholinergic, pharmacoresistance, epileptogenesis

## Abstract

Background/Objective: Status epilepticus (SE) is a life-threatening condition that requires immediate response to effectively control. Although benzodiazepines are the first-line treatment against SE, when treatment is delayed, benzodiazepine pharmacoresistance develops. In preclinical models of benzodiazepine refractory SE, the addition of antiseizure medications (ASMs) as adjunct to midazolam to reduce neuronal excitability and enhance inhibitory function is essential to protect against the neurodegeneration and epileptogenesis that follows prolonged seizure. Brivaracetam is a recently FDA-approved ASM to treat partial onset seizures in pediatric and adult patients as a monotherapy or adjunct therapy. We evaluated the potential of brivaracetam as monotherapy or in combination with midazolam and ketamine for efficacy against organophosphorus nerve agent (OPNA)-induced refractory SE in rats. Methods: Adult male rats were exposed to a seizure-inducing dose of soman and treated with atropine sulfate and the oxime asoxime chloride one minute after soman exposure and with brivaracetam alone or in combination with midazolam and ketamine 40 min after seizure onset. Multiple metrics of protection such as seizure severity, spontaneous recurrent seizure (SRS), neuronal loss, and neuroinflammation were evaluated. Results: Although brivaracetam monotherapy resulted in 100% survival, protection from the development of SRS and neurodegeneration only occurred when brivaracetam was administered as an adjunct to ketamine and midazolam. Initial seizure severity was also reduced by the combination of brivaracetam–midazolam–ketamine over monotherapy. Conclusions: Although further research is needed to determine optimal drug combinations, these preclinical findings provided further evidence that simultaneous polytherapy with ASMs improves OPNA-induced seizure outcomes.

## 1. Introduction

The development of status epilepticus (SE) may lead to self-sustaining seizures that are difficult to control and life-threatening [reviewed in [1]]. This may result in glutamatergic excitation, epileptogenesis, neurodegeneration, and death. An effective preclinical method of inducing SE is through exposure of rodents to an organophosphorus nerve agent (OPNA); OPNAs inhibit the enzyme acetylcholinesterase and lead to an excess buildup of the neurotransmitter acetylcholine, followed by glutamatergic excitation [2]. The current treatment paradigm for exposure to an OPNA includes an oxime to reactivate acetylcholinesterase (e.g., Pralidoxime, 2-PAM), an anticholinergic (e.g., atropine), and a benzodiazepine (e.g., midazolam or diazepam) to counter seizure activity [3]. The duration of seizure is associated with the extent of neurodegeneration–prompt seizure control is vital to increase survival and reduce brain damage [4]. In preclinical studies, early treatment with benzodiazepines increases survival and terminates seizure; however, when treatment is delayed to 30 min or more after exposure, prolonged seizure occurs and treatment efficacy decreases [5,6,7]. These preclinical models are clinically relevant in the case of a mass casualty civilian exposure to an OPNA, in which immediate treatment is unlikely and the identity of toxic chemical exposure is unknown. Preclinical studies in mice and rats demonstrated that the combination of the NMDA antagonist ketamine with a benzodiazepine reduced cholinergic-induced SE, even at delayed time points [8,9,10,11,12,13,14,15]. However, the neuroprotection is often incomplete, suggesting the need for a third antiseizure medication (ASM) and discovery of adjunct polytherapy treatment options that provide more complete protection against refractory SE.

Brivaracetam is a recently FDA-approved monotherapy and adjunct ASM used to treat focal seizures in adults and children [reviewed in [16]]. Brivaracetam is a selective ligand that binds to transmembrane synaptic vesicle protein 2A (SV2A) and exhibits antiseizure effects by reducing neuronal excitability and decreasing synaptic transmission [17,18]. It was proposed that SV2A helps regulate presynaptic calcium levels [19] and plays a role in exocytosis and vesicle trafficking [20]. During SE, calcium influx primarily via ionotropic glutamate receptors activates signaling cascades that are thought to lead to rapid internalization of synaptic GABA_A_ receptors (GABA_A_R) [reviewed in [21]]. Pharmacoresistance may result in part from rapid internalization of GABA_A_R, which reduces inhibition, as well as trafficking of NMDA receptors (NMDAR) to synaptic surfaces, which increases excitation and exacerbates seizure [reviewed in [21,22]]. Levetiracetam is another SV2A ligand that is FDA-approved for focal, myoclonic, and tonic–clonic seizures [17]. Compared to levetiracetam, brivaracetam exhibits faster penetration across the blood–brain barrier due to its higher lipophilicity [23], higher affinity for SV2A [24], and a faster onset of action [23].

Brivaracetam has few adverse drug–drug interactions due to its limited interactions with metabolizing enzymes and drug transporters [25]. When studied as an early treatment for SE in humans, it was found that intravenous brivaracetam exhibits a high response rate when given at a higher dosage [26]. In a pooled dataset of long-term adjunctive brivaracetam usage in humans being treated for focal seizures, brivaracetam was found to be well tolerated with relatively minor adverse effects [27]. Following electrical perforant path stimulation in rats, acute treatment with a low-dose brivaracetam–diazepam dual therapy and a high-dose brivaracetam monotherapy reduced self-sustaining SE and spontaneous recurrent seizures (SRS) [28]. This suggests potential synergistic interactions between benzodiazepines and brivaracetam. At therapeutic concentrations, brivaracetam does not modulate inhibitory and excitatory ligand-gated receptors, including GABA_A_Rs and NMDARs [29], so a midazolam–ketamine–brivaracetam triple therapy combination may be more efficacious than monotherapy.

With the potential threat of a large-scale civilian exposure, the development of a more robust antiseizure treatment to supplement current medical treatments is vital for improved protection against SE. While the addition of ketamine offers partial protection, adding an adjunctive treatment to supplement and achieve improved patient outcomes is of interest. Brivaracetam, effective clinically for focal seizures [reviewed in [16]] and promising in preclinical SE models [28], was evaluated in this study as both a monotherapy and polytherapy with midazolam and ketamine in a delayed treatment rodent model of SE. We hypothesize that a triple therapy combination consisting of midazolam, ketamine, and brivaracetam will protect against OPNA-induced SE, epileptogenesis, and neurodegeneration compared to midazolam or brivaracetam monotherapies, through the induction of a broader range of targets.

## 2. Materials and Methods

### 2.1. Animals

Adult male Sprague Dawley rats (275–300 g; Charles River Laboratories, Kingston, NY, USA) were pair-housed until the day of surgery and then individually housed for the remainder of the study. Rats were weighed daily, maintained on a 12 h:12 h light–dark cycle, (lights on at 0600), and given *ad libitum* access to food and water. The experimental protocol was approved by the Animal Care and Use Committee at the United States Army Medical Research Institute of Chemical Defense, an AAALAC accredited facility, and all procedures were conducted in accordance with the principles stated in the Guide for the Care and Use of Laboratory Animals (National Research Council, 2011), the Public Health Service Policy on Humane Care, and Use of Laboratory Animals, and the Animal Welfare Act of 1966 (P.L. 89-544), as amended.

### 2.2. Telemetry Transmitter Implantation for Electroencephalographic (EEG) Activity Recording

Telemetry devices (F40-EET or HD-S02; Data Sciences International; DSI; St. Paul, MN, USA) for continuous EEG recording were implanted subcutaneously (SC) in the rats using surgical procedures described previously [8]. Prior to surgery, the rats were pretreated with meloxicam (1 mg/kg; SC) and anesthetized with isoflurane (5% induction, 2–5% maintenance). The rats were secured in a Kopf stereotaxic apparatus (David Kopf Instruments, Tujunga, CA, USA) and four stainless steel screw electrodes were implanted cortically through the skull 2 mm from each side of the midline at 1.6 mm anterior and 4 mm posterior to bregma. Stainless steel wires extending from the transmitters were tunneled SC with the ends of wires wrapped around each screw electrode. Self-curing dental acrylic (Ortho-Jet™, Lang Dental Manufacturing Company, Inc., Wheeling, IL, USA) was used to secure the wire-wrapped electrodes to the skull. Immediately following removal from anesthesia, buprenorphine-sustained release (SR; 1.2 mg/kg, SC) was administered as a post-operative analgesia and rats were given 7–10 days surgical recovery prior to soman exposure.

### 2.3. Soman Exposure and Administration of Therapeutics

On exposure day, rats were exposed SC to a seizure-inducing dose of soman (GD; 118.1 µg/kg; 0.5 mL/kg; US Army Combat Capabilities Development Command Chemical Biological Center, Aberdeen Proving Ground, MD, USA). This dose of soman is approximately equivalent to an LD_90_; therefore, an untreated soman control group was not included in this study due to expected lethality. A subset of rats received SC saline instead of soman for control comparisons in body weights and neuropathology. One min later, soman-exposed rats were treated with an admix of atropine sulfate (ATS; 2 mg/kg, IM; Sigma-Aldrich, St. Louis, MO, USA) and asoxime chloride (HI-6 DMS; 118.5 mg/kg, IM; synthesized by Kalexyn Medicinal Chemistry, Kalamazoo, MI, USA) to increase survival. Seizure onset was identified in real time by a blind observer monitoring EEG recordings and was defined as rhythmic, high amplitude spikes (>2 × baseline values) lasting at least 10 s. Subjects that seized following exposure received midazolam (3 mg/kg, IP; Hospira, Lake Forest, IL, USA), brivaracetam (UCB Inc., Smyrna, GA, USA; 10, 30, or 100 mg/kg, IP), midazolam (3 mg/kg, IP) and brivaracetam (10 mg/kg, IP), or a combination of midazolam (3 mg/kg, IP), brivaracetam (10 mg/kg, IP), and ketamine (30 mg/kg, IP; Zoetis, Parsippany-Troy Hills, NJ, USA) 40 min after seizure onset. Midazolam and ketamine were diluted in sterile water.

Percentage of survival was calculated at the study endpoint, 14 days from soman exposure. Rats that did not survive to the study endpoint were excluded from SRS, neuropathology, and body weights analyses; rats that did not survive exposure day were excluded from acute seizure, power, and temperature analyses. Subjects exposed to soman that did not seize were not treated and were excluded from data analysis.

Observers blinded to treatment monitored rats continuously for 3 h following exposure and every 30 min until close of business for manual scoring of behavior based on a modified Racine scale [30]. Behavioral seizures were scored according to five stages: (1) masticatory movements, (2) head myoclonus, (3) limb clonus and/or tonus, (4) forelimb clonus with rearing, and (5) rearing and falling and/or tonic–clonic convulsions. At the conclusion of the observation period, sterile saline (5 mL, SC) was administered. Additional saline administration and food pellets dissolved with sugar and water were given daily to aid with recovery.

### 2.4. EEG Seizure Identification

At least 24 h prior to exposure, each home cage was placed over an RPC-1 PhysioTel receiver (DSI, Inc., St. Paul, MN, USA). Continuous acquisition of EEG and temperature data until the study endpoint (14 days from soman exposure) was recorded with Dataquest ART Acquisition software v 4.3 for subjects with F40-EET transmitters and Ponemah software v6.41 (DSI, Inc.) for subjects with HD-S02 transmitters. At least 24 h of baseline data was recorded prior to exposure.

EEG recordings were scored by a reviewer blinded to treatment groups for seizure activity following previously established methods [8,31]. For spectral analysis, the EEG power spectrum was divided into five frequency bands: delta (0.1–4.0 Hz), theta (4.1–8.0 Hz), alpha (8.1–12 Hz), beta (12.1–25 Hz), and gamma (25.1–50 Hz). The mean power was calculated for each band and integrated in 10 min bins. Seizure severity was quantified using the EEG power integral, calculated across the full 0.1–100 Hz spectrum. A custom MATLAB vr2017b algorithm computed hourly average power spectra and converted these values to decibels/h using the following formula: [decibels = 10 × (Log(V^2^sample/V^2^baseline))] × 60 min. This approach allowed for the comparison of EEG power in specific time periods after seizure onset or treatment relative to baseline.

### 2.5. Neuropathology Assessment

At the study endpoint, surviving subjects were administered pentobarbital sodium (Euthasol; Virbac or Fatal-Plus; Patterson Veterinary, Loveland, CO, USA) and perfused first with 0.9% heparinized saline in 0.1 M phosphate buffer, followed by 4% paraformaldehyde (FD Neurotechnologies, Columbia, MD, USA). Brains were removed, post-fixed in 4% paraformaldehyde for 6 h at 4–8 °C, cryoprotected for up to one week in 20% sucrose in phosphate buffer, then rapidly frozen for storage at −75 °C. FD Neurotechnologies, Inc. (Columbia, MD, USA) performed tissue sectioning and staining according to methods described in Hsu et al. [32]. Coronal sections (30 µm) were histochemically labeled for neuronal nuclear protein (NeuN; mouse anti-NeuN IgG 1:10,000; Millipore, Billerica, MA, USA) and ionized calcium-binding adaptor molecule-1 (Iba1; rabbit anti-Iba1 IgG 1:8000; Wako Chemicals, Richmond, VA, USA). A cresyl violet counterstain was utilized in Iba1-stained tissue for enhanced visualization of anatomical landmarks.

Stained tissue sections were permanently mounted on slides with a coverslip. An Olympus BX61IVS microscope (Olympus Corporations of the Americas, Center Valley, PA, USA) with a Pike F-505 camera (Allied-Vision, Exton, PA, USA) was used to scan slides and capture images. NeuN-stained sections were scanned at 10× and Iba1-stained sections were scanned at 20× magnification. Regions of interest for NeuN-stained slides included the basolateral amygdala, layer 3 of the piriform cortex, lateral thalamus, medial thalamus, and CA1 of the hippocampus. Sections stained with NeuN were analyzed in ImagePro v7.0 (Media Cybernetics, Inc., Rockville, MD, USA). Contrast was inverted for better visualization of NeuN+ cells. Cell density values were obtained using automated counts of NeuN+ cells in the piriform, amygdala, and thalamus regions. Stereological counts of the rat hippocampus CA1 were completed using an Olympus BX63 microscope (Olympus Corporations of the Americas, Center Valley, PA, USA) equipped with MBF Stereoinvestigator software v2024.13, using a 100× oil immersion objective, and a 250 × 250 grid size, with 40 × 40 counting frame. Estimated population using number weighted section thickness was acquired and indexed against the total area of the traced section to give an estimated cell density. The Gundersen Coefficient of error was kept below 0.1 for all estimated counts. Slides stained with Iba1 were analyzed for Iba1+ cell density and cell body-to-size ratio in ImageJ v1.54d (National Institutes of Health; NIH, Bethesda, MD, USA) to evaluate microglial activation in layer 3 of the piriform cortex, basolateral amygdala, medial thalamus, lateral thalamus, and CA1 region of the hippocampus using modified methods [33,34], as described in Marrero Rosado et al. [35]. Cell body-to-size ratio indicates any microglial morphological changes and/or activation and Iba1+ cell density measures the level of microglial proliferation to a specific region.

### 2.6. Data Analysis

Data analysis was conducted using GraphPad Prism v10.2.3 (GraphPad Software Boston, MA, USA), except for the repeated measures paradigm, which was conducted in SPSS v28 (IBM Inc. Armonk, NY, USA). Graphs were generated using GraphPad Prism v10.2.3 (GraphPad Software Boston, MA, USA). G*Power (Version 3.1.9.7, Heinrich-Heine-Universität Düsseldorf, Düsseldorf, Germany) was used to confirm that the study design (6–10 animals/group) achieved sufficient power assuming an alpha of 0.05. General linear model analyses with a repeated measures paradigm were used to determine the effect of treatment type on body temperature, body weight, and EEG power spectral density. To determine the relationship between different treatment groups and the percentage of animals that developed SRS by the study endpoint, a binary logistic regression analysis and contingency table analysis using chi-square test were performed. A Kaplan–Meier analysis was performed to determine the effect of treatment on latency to SRS onset. A Kruskal–Wallis test was used to analyze the effect of treatment on the number of SRS occurrences. Ordinal data obtained from behavioral observations based on modified Racine scale were analyzed with a Kruskal–Wallis test. Seizure duration was analyzed using a one-way ANOVA followed by Dunnett’s one-sided *t*-test to compare brivaracetam monotherapy and combination therapies against midazolam. For comparison of NeuN+ cell density, Iba1+ cell density, and Iba1+ cell body-to-size ratio, a one-way ANOVA was performed followed by Dunnett’s one-sided *t*-test separately for each brain region to compare brivaracetam therapies against midazolam and control groups.

## 3. Results

### 3.1. Survival, Behavioral Seizure, Body Temperature, and Body Weight

**Figure 1:**
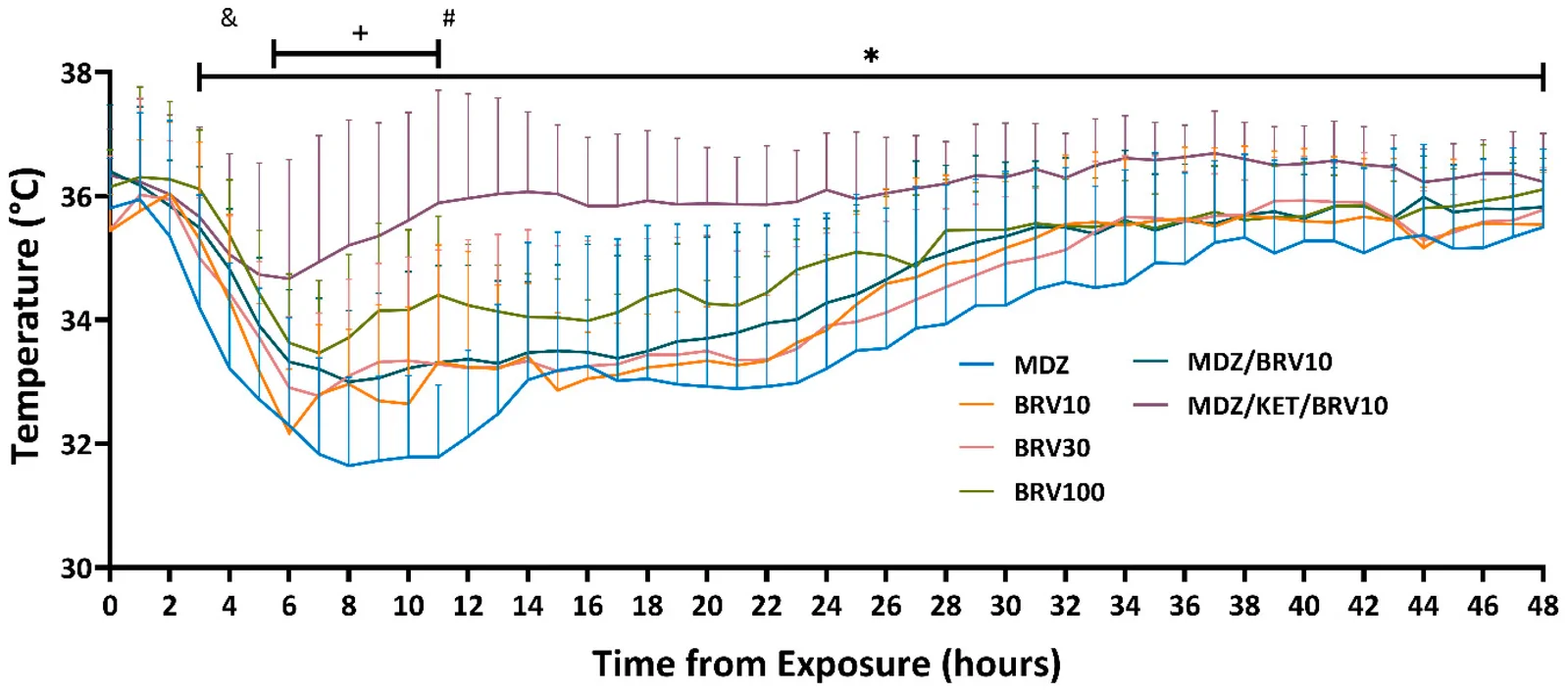
The effect of brivaracetam monotherapy and combination therapies on body temperature in adult male rats exposed to soman. Rats were exposed to a seizure-inducing dose of soman followed by midazolam only (MDZ), brivaracetam only (BRV at 10, 30, or 100 mg/kg), midazolam–brivaracetam dual therapy (MDZ/BRV10), or midazolam–ketamine–brivaracetam triple therapy (MDZ/KET/BRV at 10 mg/kg) 40 min after seizure onset. Temperature recordings in degrees Celsius (°C) averaged in 1 h time bins are displayed as mean ± SD from exposure to 48 h after exposure. Within the first few hours after GD exposure, all soman-exposed rats experienced a drop in average temperature from baseline levels. Compared to MDZ, the MDZ/KET/BRV10 and BRV100 groups had significantly higher temperature from 3 to 48 h and 5 to 11 h from exposure, respectively. * *p* < 0.05 MDZ/KET/BRV10 compared to MDZ; # *p* < 0.05 BRV10 compared to MDZ. & *p* < 0.05 BRV30 compared to MDZ; + *p* < 0.05 BRV100 compared to MDZ. MDZ *n* = 7, BRV10 *n* = 8, BRV30 *n* = 8, BRV100 *n* = 6, MDZ/BRV10 *n* = 7, MDZ/KET/BRV10 *n* = 11.

No significant difference in percentage of survivors at the study endpoint was observed. Rats treated with only midazolam had an 87.5% survival rate (*n* = 7/8) while rats treated with brivaracetam (10, 30, and 100 mg/kg) all had 100% survival (*n* = 8/8, *n* = 8/8, and *n* = 6/6 respectively). The rats treated with the dual therapy had 70% survival (*n* = 7/10) and the triple therapy had 71.4% survival (*n* = 10/14). One rat in the triple therapy group was euthanized 4 days after exposure for humane endpoint and was excluded from SRS and neuropathological analysis. As observed previously after soman exposure in rats [36], a transient drop in body temperature was observed in all rats within a few hours following soman exposure. However, rats treated with 100 mg/kg brivaracetam and the triple therapy had significantly higher body temperature compared to midazolam for 5 to 11 h and 3 to 48 h from exposure, respectively (Figure 1). All OPNA-exposed rats exhibited a 10–20% decrease in body weight compared to control one day after exposure. On day 2 from exposure, rats that received midazolam, brivaracetam (30 mg/kg and 100 mg/kg), or the triple therapy were not significantly different from controls. Rats in the brivaracetam (10 mg/kg) and dual therapy groups recovered weight back to control levels by day 6.

### 3.2. Seizure Activity and Epileptogenesis

**Figure 2:**
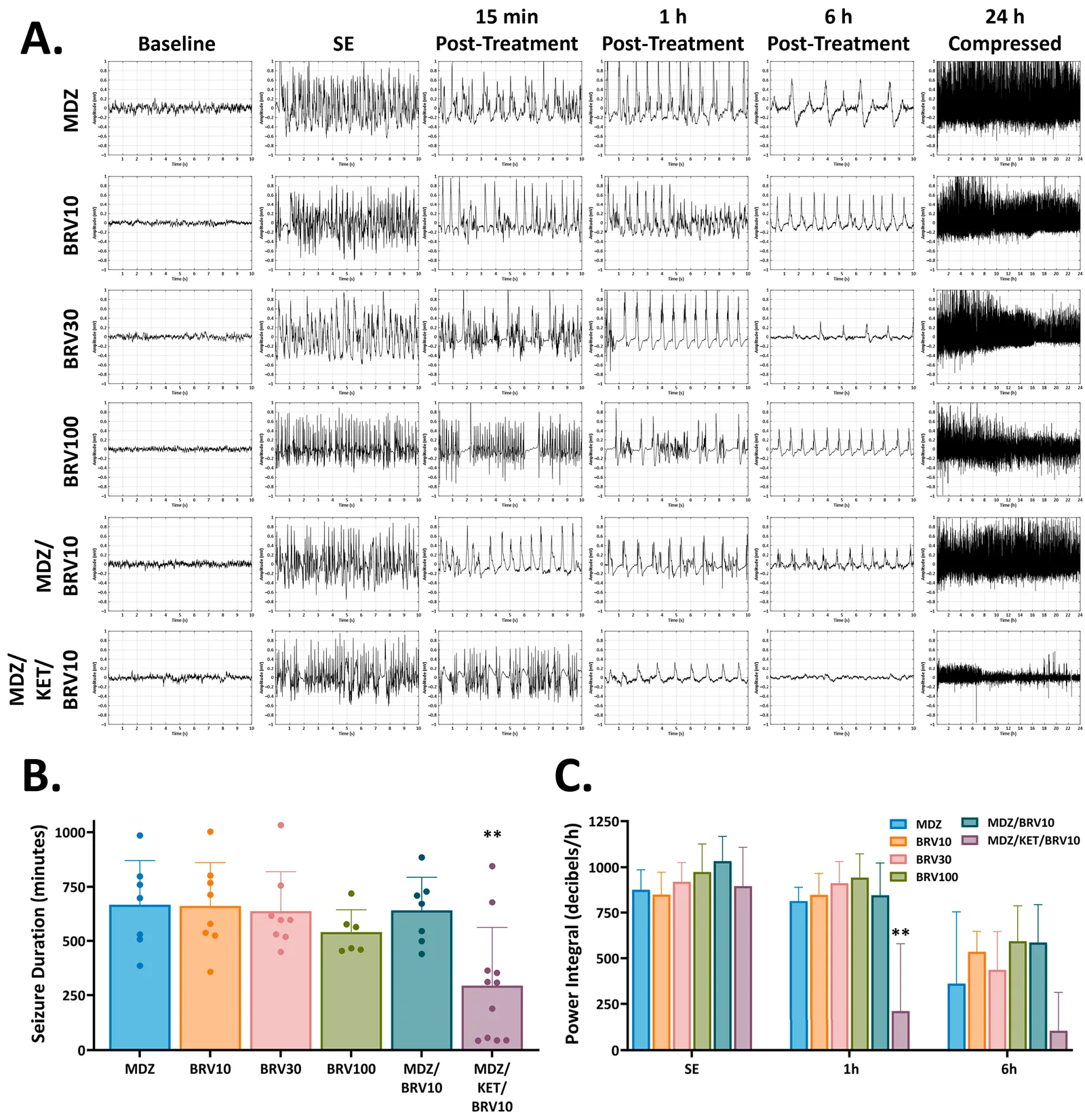
The effect of brivaracetam treatment combinations on the initial seizure duration and power integral following GD exposure in adult male rats. Rats exposed to GD were treated with midazolam monotherapy (MDZ), brivaracetam monotherapy (BRV at 10, 30, or 100 mg/kg), midazolam–brivaracetam dual therapy (MDZ/BRV10), or polytherapy with midazolam, ketamine, and brivaracetam (MDZ/KET/BRV at 10 mg/kg) 40 min after seizure onset. (**A**) Representative EEG tracings in 10 s intervals are shown at baseline, during status epilepticus (SE), 15 min after treatment, 1 h after treatment, and 6 h after treatment, and in a compressed 24 h interval. (**B**) Seizure duration was reduced in rats treated with MDZ/KET/BRV10 compared to rats treated with MDZ. (**C**) At 1 h from treatment, power integral was reduced in rats treated with MDZ/KET/BRV10 compared to MDZ. Data are shown as mean ± SD; ** *p* < 0.01 compared to MDZ. MDZ *n* = 7, BRV10 *n* = 8, BRV30 *n* = 8, BRV100 *n* = 6, MDZ/BRV10 *n* = 7, MDZ/KET/BRV10 *n* = 11.

**Figure 3:**
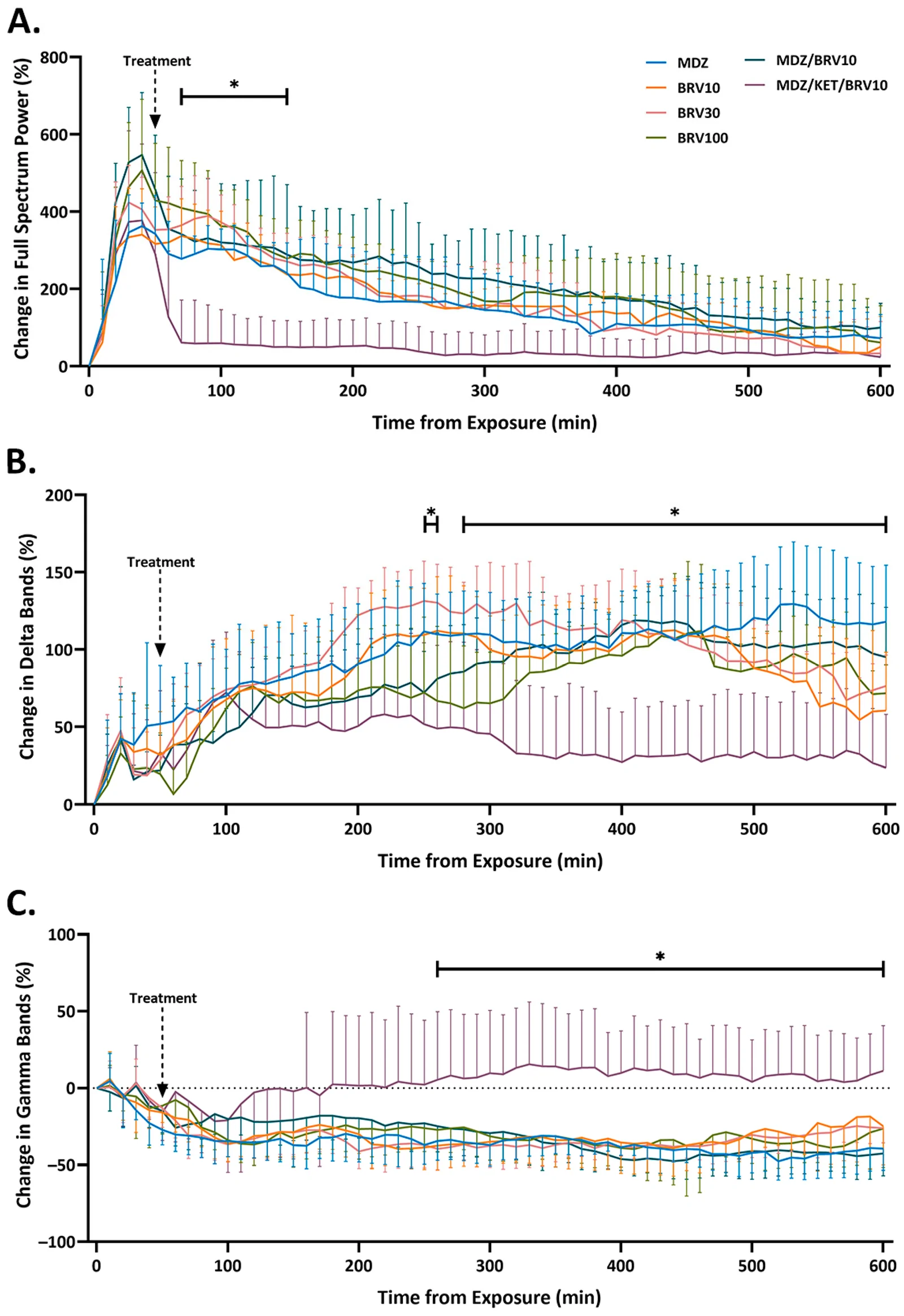
The effect of brivaracetam treatment combinations on power spectra analysis following GD exposure in adult male rats. Rats exposed to GD were treated with midazolam monotherapy (MDZ), brivaracetam monotherapy (BRV at 10, 30, or 100 mg/kg), midazolam–brivaracetam dual therapy (MDZ/BRV10), or polytherapy with midazolam, ketamine, and brivaracetam (MDZ/KET/BRV at 10 mg/kg) 40 min after seizure onset. The percent of relative change from baseline in the power (shown as mean ± SD) of (**A**) full spectrum, (**B**) delta, and (**C**) gamma bands was calculated for each treatment group and averaged in 10 min bins. The MDZ/KET/BRV10 group had reduced change in full spectrum power compared to MDZ 70–150 min after exposure. The MDZ/KET/BRV10 group attenuated the effects of soman on delta band 250–260 and 280–600 min after exposure and on gamma band 250–600 min after exposure compared to MDZ. * *p* < 0.05 MDZ/KET/BRV10 compared to MDZ. MDZ *n* = 7, BRV10 *n* = 8, BRV30 *n* = 8, BRV100 *n* = 6, MDZ/BRV10 *n* = 7, MDZ/KET/BRV10 *n* = 11.

Representative EEG tracings are shown at baseline, during status epilepticus (SE), 15 min post-treatment, 1 h post-treatment, 6 h post-treatment, and in a compressed 24 h interval in Figure 2A. The midazolam–ketamine–brivaracetam treatment group had reduced seizure duration (Figure 2B) and EEG power integral at 1 h (Figure 2C) compared to midazolam monotherapy. The rats treated with the triple therapy had reduced behavioral seizure 100 to 130 min after exposure compared to midazolam. The midazolam–ketamine–brivaracetam triple therapy showed reduced percent change in full spectrum power compared to midazolam 70 to 150 min from exposure (Figure 3A). Soman exposure increased delta band activity and reduced gamma band activity from baseline in all rats within a few minutes of exposure. The midazolam–ketamine–brivaracetam therapy significantly attenuated the effects of soman on delta band activity 250 to 260 and 280 to 600 min from exposure (Figure 3B) and on gamma band activity 250 to 600 min from exposure (Figure 3C) compared to midazolam monotherapy.

**Figure 4:**
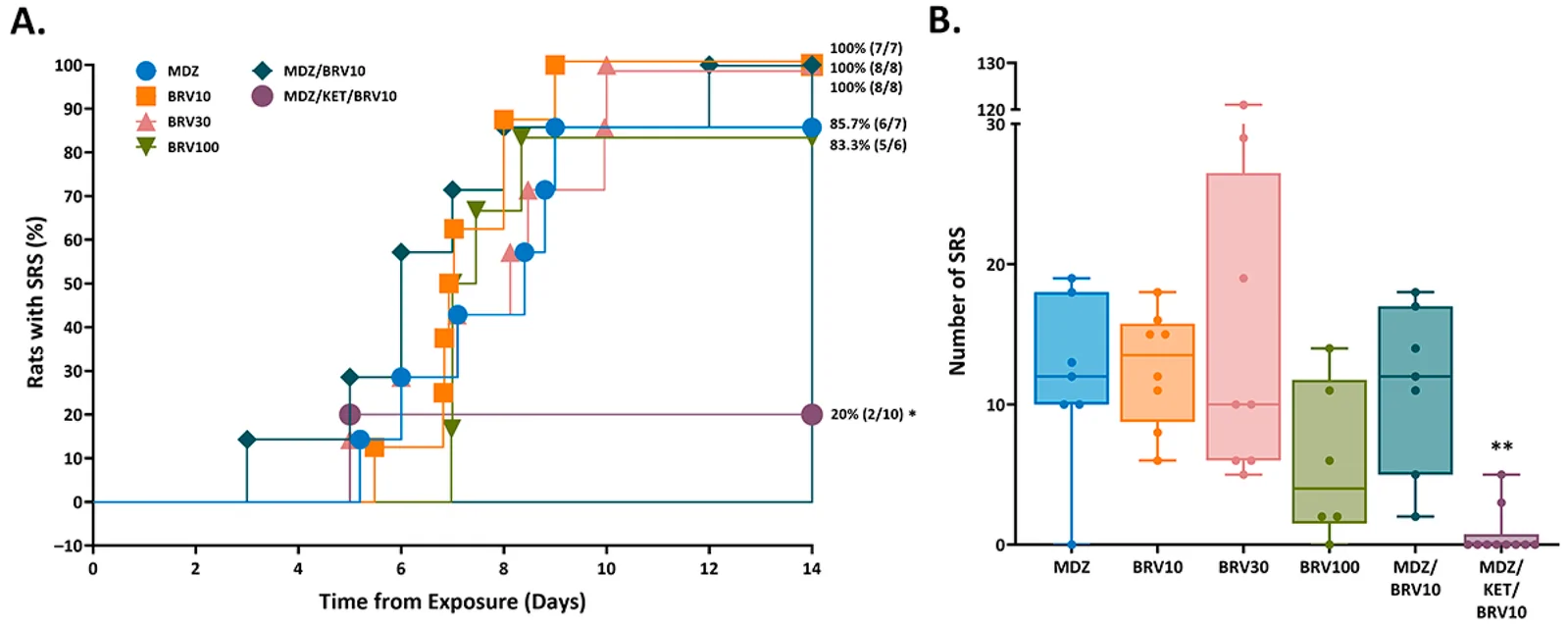
The effect of brivaracetam treatment combinations on the development of spontaneous recurrent seizures (SRS) following GD exposure in adult male rats. Rats exposed to GD were treated with midazolam monotherapy (MDZ), brivaracetam monotherapy (BRV at 10, 30, or 100 mg/kg), midazolam–brivaracetam dual therapy (MDZ/BRV10), or polytherapy with midazolam, ketamine, and brivaracetam (MDZ/KET/BRV at 10 mg/kg) 40 min after seizure onset. SRS incidence and count were assessed 14 days after exposure. (**A**) Fewer soman-exposed rats treated with MDZ/KET/BRV10 developed SRS by the study endpoint compared to rats treated with MDZ. (**B**) The MDZ/KET/BRV10 group had fewer occurrences of SRS than MDZ. The median (±IQR) number of SRS occurrences is shown. * *p* < 0.05 and ** *p* < 0.01 compared to MDZ. MDZ *n* = 7, BRV10 *n* = 8, BRV30 *n* = 8, BRV100 *n* = 6, MDZ/BRV10 *n* = 7, MDZ/KET/BRV10 *n* = 10.

The percentage of rats that developed SRSs and total number of SRSs were evaluated in all OPNA-exposed rats 14 days from exposure. It was observed that the midazolam–ketamine–brivaracetam triple therapy group reduced epileptogenesis. A Kaplan–Meier analysis revealed a significant group effect on latency to SRS onset between the triple therapy and midazolam monotherapy (Figure 4A). The triple therapy group had a significantly lower percentage of rats that develop SRS 14 days after exposure (Figure 4A) and fewer SRS occurrences compared to midazolam (Figure 4B).

### 3.3. Neuropathology Resulting from Cholinergic-Induced Status Epilepticus

**Figure 5:**
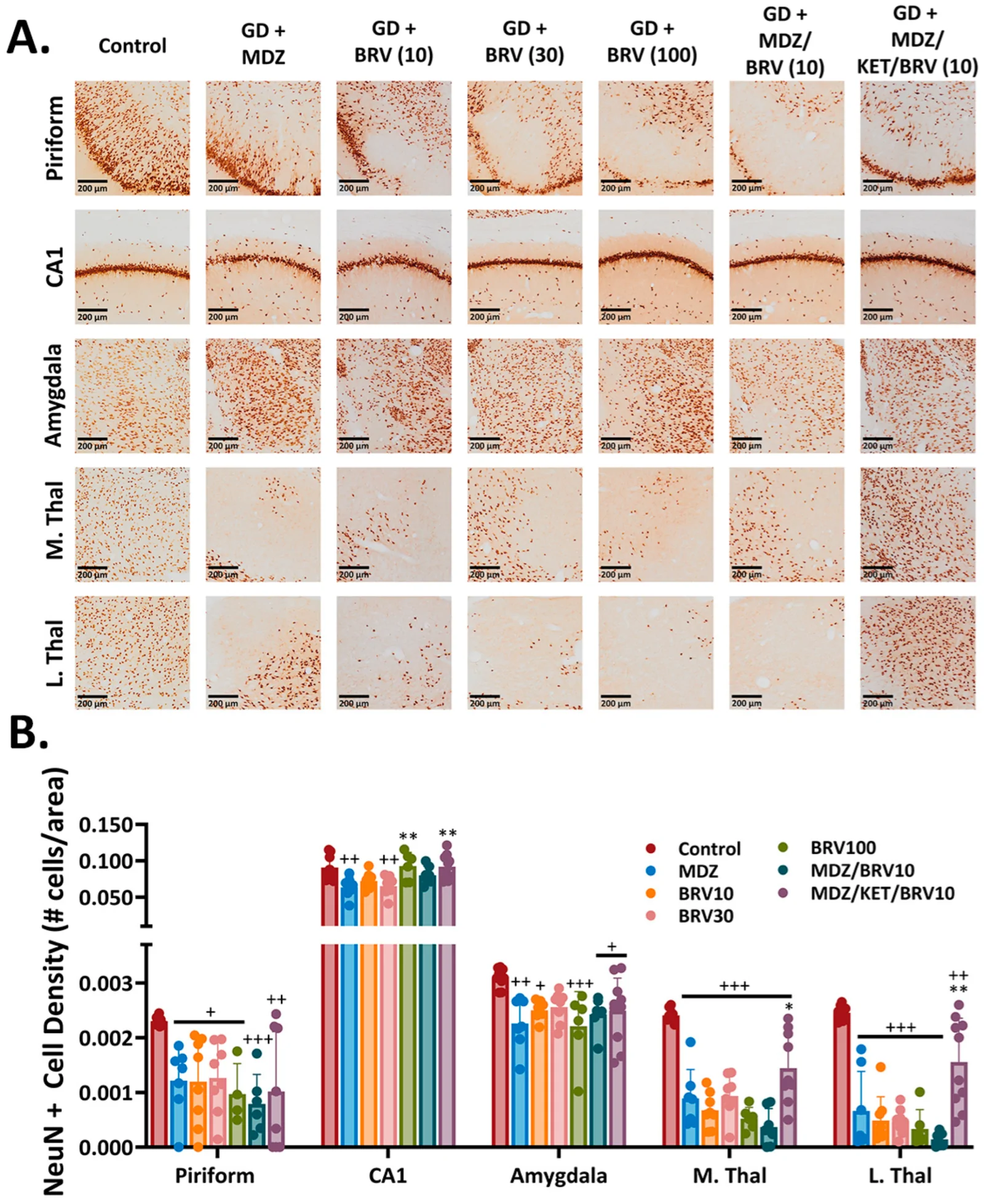
The effect of brivaracetam treatment combinations on neuronal loss induced by status epilepticus in adult male rats. Rats exposed to GD were treated with midazolam monotherapy (MDZ), brivaracetam monotherapy (BRV at 10, 30, or 100 mg/kg), midazolam–brivaracetam dual therapy (MDZ/BRV10), or polytherapy with midazolam, ketamine, and brivaracetam (MDZ/KET/BRV at 10 mg/kg) 40 min after seizure onset. Control rats were not exposed to GD. (**A**) NeuN-stained coronal images are shown of the piriform cortex (layer 3), CA1 of the hippocampus, amygdala, medial thalamus (M. Thal), and lateral thalamus (L. Thal). Images were taken at 10× magnification. Scale bar = 200 µm. (**B**) MDZ/KET/BRV10 and BRV100 prevented neuronal loss in the CA1. Compared to MDZ, MDZ/KET/BRV10 also reduced neuronal loss in the M. Thal. and L. Thal. None of the treatments protected against neuronal loss in the piriform, M. Thal., and L. Thal compared to controls. Only BRV30 was not significantly different from controls in the amygdala. Data are shown as mean ± SD. + *p* < 0.05, ++ *p* < 0.01, and +++ *p* < 0.001 compared to Control; * *p* < 0.05 and ** *p* < 0.01 compared to MDZ. Control *n* = 9, MDZ *n* = 7, BRV10 *n* = 8, BRV30 *n* = 8, BRV100 *n* = 6, MDZ/BRV10 *n* = 7, MDZ/KET/BRV10 *n* = 10.

Neuropathological analysis was evaluated in brain regions notably targeted by SE [7,35]. The NeuN+ stain was used to assess neuronal loss in the piriform cortex, CA1 of the hippocampus, amygdala, lateral thalamus, and medial thalamus, as imaged in Figure 5A. Both the triple therapy and 100 mg/kg brivaracetam prevented neuronal loss in the CA1, with NeuN+ cell densities greater than midazolam and not significantly different from unexposed controls (Figure 5B). The triple therapy, but not brivaracetam monotherapy, also reduced neuronal loss in the medial and lateral thalamus compared to midazolam. However, none of the therapies protected against neuronal loss in the piriform, medial thalamus, and lateral thalamus compared to controls. In the amygdala, the 30 mg/kg brivaracetam group showed NeuN+ cell densities that were not significantly different from controls; however, no additional protection was observed in the amygdala across other treatment groups or compared to midazolam.

**Figure 6:**
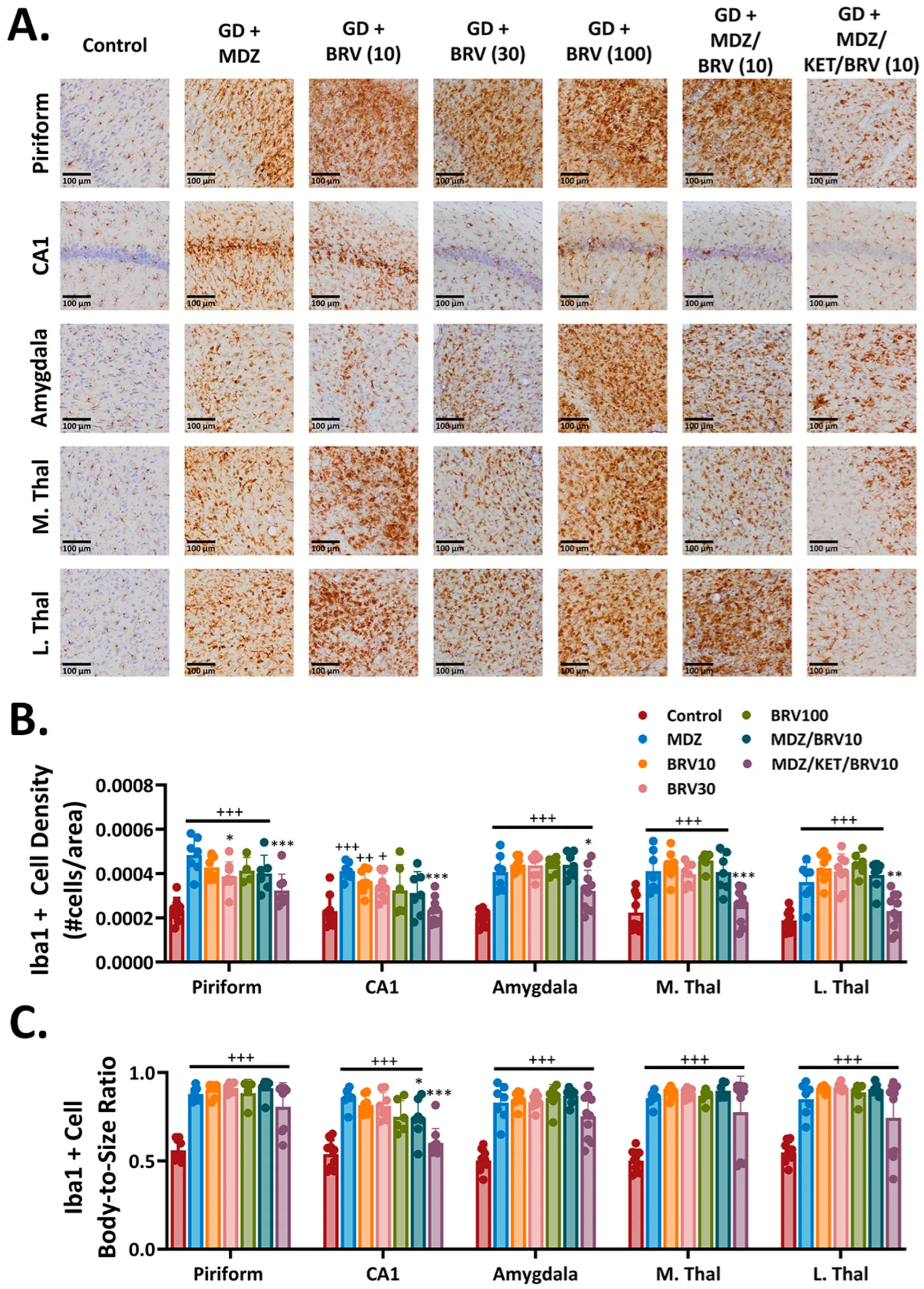
The effect of brivaracetam and midazolam treatment combinations on neuroinflammation following status epilepticus in adult male rats. Rats exposed to GD were treated with midazolam monotherapy (MDZ), brivaracetam monotherapy (BRV at 10, 30, or 100 mg/kg), midazolam–brivaracetam dual therapy (MDZ/BRV10), or polytherapy with midazolam, ketamine, and brivaracetam (MDZ/KET/BRV at 10 mg/kg) 40 min after seizure onset. (**A**) Iba1-stained coronal images are shown of the piriform cortex (layer 3), CA1 of the hippocampus, amygdala, medial thalamus (M. Thal), and lateral thalamus (L. Thal). Images were taken at 20× magnification. Scale bar = 100 µm. (**B**) Compared to MDZ, MDZ/KET/BRV reduced Iba1+ cell density in all brain regions, and only had increased density in the amygdala compared to controls. BRV30 also reduced Iba1+ density in the piriform compared to MDZ. Rats treated with MDZ, BRV, or MDZ/BRV10 experienced a greater Iba1+ cell density in the piriform, amygdala, M. Thal, and L. Thal compared to unexposed controls. (**C**) MDZ/BRV10 and MDZ/KET/BRV10 reduced Iba1+ cell body-to-size ratio in the CA1 compared to MDZ. Compared to controls, Iba1+ body-to-size ratio was increased in the piriform, amygdala, M. Thal., and L. Thal. across all treatment groups. Data are shown as mean ± SD. + *p* < 0.05, ++ *p* < 0.01, and +++ *p* < 0.001 compared to Control; * *p* < 0.05, ** *p* < 0.01, and *** *p* < 0.001 compared to MDZ. Control *n* = 9, MDZ *n* = 7, BRV10 *n* = 8, BRV30 *n* = 8, BRV100 *n* = 6, MDZ/BRV10 *n* = 7, and MDZ/KET/BRV10 *n* = 10.

Analysis of Iba1-stained tissue, as shown in Figure 6A, was used for the quantification of Iba1+ cell density and Iba1+ cell body-to-size ratio in the piriform cortex, CA1 of the hippocampus, amygdala, medial thalamus, and lateral thalamus. Rats treated with midazolam monotherapy, brivaracetam monotherapy, or dual therapy experienced microglial activation and proliferation in the piriform, amygdala, medial thalamus, and lateral thalamus compared to unexposed controls (Figure 6B). However, compared to midazolam, the triple therapy reduced Iba1+ cell density in all evaluated brain regions and 30 mg/kg brivaracetam reduced Iba1+ cell density in the piriform. Both the dual and triple therapy also reduced Iba1+ cell body-to-size ratio in the CA1 compared to midazolam but did not prevent soman-induced microglial activation in the other brain regions (Figure 6C).

## 4. Discussion

The present study sought to compare the efficacy of the standard of care benzodiazepine monotherapy to brivaracetam monotherapy, brivaracetam–midazolam dual therapy, and brivaracetam–ketamine–midazolam triple therapy in a delayed treatment model of soman-induced SE in rats. The addition of brivaracetam as a third antiseizure medication reduced seizure duration, seizure severity, epileptogenesis, neurodegeneration and neuroinflammation compared to midazolam monotherapy in soman-exposed rats. While none of the brivaracetam treatment groups were fully neuroprotective and there were no significant differences in survival outcome, it is notable that all rats who received a brivaracetam monotherapy survived until the study endpoint. However, the brivaracetam monotherapy failed to reduce acute seizure and SRS, whereas the midazolam–ketamine–brivaracetam triple therapies reduced both measures compared to midazolam.

A neuroinflammatory response takes place following soman-induced SE, mediated by the activation of glial cells, including microglia [10,37,38]. Additionally, high levels of brivaracetam were discovered to produce mild neuroinflammation in vitro [39]. In rats, an increase in microglial cell density in the hippocampus, bilateral amygdala, endopiriform nucleus, paraventricular thalamic nucleus, and the left piriform cortex was also observed six weeks after kainic acid-induced status epilepticus with oral treatment of brivaracetam, but not levetiracetam [40]. Our data showed that although neuroinflammation was observed in the brivaracetam monotherapy (10, 30, and 100 mg/kg) and midazolam–brivaracetam (10 mg/kg) dual therapy groups following soman exposure, midazolam–ketamine–brivaracetam triple therapy prevented an increase in microglial proliferation in the piriform, CA1, medial thalamus, and lateral thalamus. This suggests that the addition of ketamine may be responsible for the reduced neuroinflammatory response seen only in the triple therapy, as previous studies have also observed an anti-inflammatory effect of ketamine as an adjunct ASM in soman-induced SE [10,37].

The midazolam–ketamine–brivaracetam triple therapy provided neuroprotection in the CA1 of the hippocampus, with a significantly higher neuronal cell density and reduced microgliosis compared to midazolam. The hippocampus is a target of SE-induced damage [2,41,42], and hippocampal damage has been further linked to the occurrence of SRS [41,43]; reviewed in [44]. It has also been suggested that neuroinflammation plays a role in epileptogenesis and the development of SRS [45]. Therefore, it is possible the neuroprotective effect of the triple therapy in the hippocampus and reduced neuroinflammation may relate to the reduction in SRS only seen in this treatment group. In addition to the protection of the CA1 in rats treated with triple therapy, the medial and lateral thalamus were also protected compared to midazolam-only treated rats. Neuroprotection provided by treatment with triple therapy was incomplete as neuronal loss was observed in the piriform, amygdala, medial thalamus, and lateral thalamus compared to controls.

The midazolam–ketamine–brivaracetam triple therapy had a lower change in gamma bands and delta bands from baseline compared to midazolam in soman-exposed rats. Seizure activity has been associated with a decrease in the gamma bands [46] and an increase in delta bands [reviewed in [47]], which is in line with the midazolam–ketamine–brivaracetam triple therapy having the shortest acute seizure. Delta band increase can also be a marker for soman-induced brain damage [reviewed in [47]]. The low percent change in the delta bands in the midazolam–ketamine–brivaracetam group is compatible with its enhanced neuroprotection reported here. Furthermore, GABA-mediated inhibition is thought to play a major role in gamma band frequency [48,49,50,51]. The increased change in gamma bands in the triple therapy compared to midazolam suggests that GABA receptors may be protected in soman-exposed rats given this treatment. Although the exact molecular mechanism has yet to be determined, there may be synergistic drug–drug interactions between midazolam, a positive modulator of GABA, with ketamine and brivaracetam to enhance GABAergic inhibition. For example, Niquet et al. [28] suggested that brivaracetam may increase the concentrations of diazepam in plasma and brain, enhancing its effectiveness.

OPNA-induced SE differs from many other chemoconvulsant seizure models because seizures originate from irreversible inhibition of acetylcholinesterase, resulting in an initial cholinergic crisis followed by glutamatergic excitation, rapid benzodiazepine pharmacoresistance and widespread neuropathology. Despite these mechanistic differences, many downstream features of OPNA-induced SE, such as prolonged seizures, neurodegeneration, neuroinflammation, and epileptogenesis, are shared with other experimental SE models [52]. In the delayed treatment model of OPNA-induced SE tested here, only the polytherapy of midazolam, ketamine, and brivaracetam reduced SRS and neuropathological changes; all other monotherapy and dual therapy combinations were insufficient. The benefit of simultaneous polytherapy over midazolam monotherapy is well-established in preclinical studies, as improved outcomes were reported with combinations of phenobarbital, valproic acid, allopregnanolone, lacosamide, rufinamide, each administered as a third antiseizure medication in combination with ketamine and midazolam [8,53,54,55]; the present study extends this list to include brivaracetam as an effective third ASM in combination with ketamine and midazolam.

Our laboratory has extensively evaluated the efficacy of midazolam–ketamine dual therapy against status epilepticus [8,9,10,11,13,14,56], but only partial protection suggests the need for a third antiseizure medication. A limitation of the current study is that a midazolam–ketamine dual therapy group was not included for direct comparison to the triple therapy. Since we previously reported incomplete protection with midazolam–ketamine dual therapy, the goal was to evaluate whether adding brivaracetam improves outcomes compared to the standard-of-care midazolam. While the triple therapy resulted in antiseizure effects compared to midazolam alone, neuroprotection was incomplete, and thus future studies are warranted to determine the most effective polytherapy combination. In conclusion, the current study suggests that brivaracetam may be beneficial as an adjunct antiseizure medication to benzodiazepines and ketamine against cholinergic-induced SE.

## Data Availability

Data may be requested from the corresponding author.

## Abbreviations

The following abbreviations are used in this manuscript:

ASM	Antiseizure Medication
OPNA	Organophosphorus Nerve Agent
SE	Status Epilepticus
SRS	Spontaneous Recurrent Seizure
SV2A	Synaptic Vesicle Protein 2A
